# Supplementary material for: Polycomb and Notch signaling regulate cell proliferation potential during Caenorhabditis elegans life cycle
Source: Life Sci Alliance. 2018 Dec 26;2(1):e201800170. doi: 10.26508/lsa.201800170 (PMC6306570; doi:10.26508/lsa.201800170)
Supplement: Supplementary file 1 [file LSA-2018-00170_TableS1.docx]

**Table S1**

| **RNAi gene:** | **functionality of the gene:** | **Reference:** |
| --- | --- | --- |
| *L4440* | vector control |  |
| *lin-12* | One of the two Notch receptors required for the Y to PDA induction and fate commitment during embryogenesis. | (Jarriault, Schwab, & Greenwald, 2008]) (I. Greenwald, 2005; I. S. Greenwald, Sternberg, & Horvitz, 1983) |
| *glp-1* | Second Notch receptor involved in cell fate commitment during embryogenesis and in the control of the mitotic cycle of germ cells | (Berry, Westlund, & Schedl, 1997; Djabrayan, Dudley, Sommermann, & Rothman, 2012; Priess, 2005) |
| *mep-1* | A homolog of the NURD complex, which is required for somatic differentiation and might counteract MES. | (Unhavaithaya et al., 2002) |
| *unc-120* | Downstream factor of *hlh-1* involved in embryonic body wall muscle development and which ectopic induction converts early embryos into muscle tissue. | (Baugh et al., 2005; Fukushige, Brodigan, Schriefer, Waterston, & Krause, 2006) |
| *lag-2* | Notch ligand. | (Lambie & Kimble, 1991; Priess, 2005) |
| *elg-27* | A member of the NODE complex required in the initiation of the Y to PDA transdifferentiation. | (Kagias, Ahier, Fischer, & Jarriault, 2012) |
| *sem-4* | A DNA-binding factor interacting with NuRD and NODE, required in the initiation of the Y to PDA transdifferentiation. | (Jarriault et al., 2008]) (Kagias et al., 2012) |
| *ceh-6* | A member of the NODE complex required in the initiation of the Y to PDA transdifferentiation. | (Kagias et al., 2012) |
| *fbf-1* | A RNA-binding protein promoting continuous mitosis in germ cells. | (Kimble & Crittenden) |
| *fbf-2* | A RNA-binding protein promoting continuous mitosis in germ cells. | (Kimble & Crittenden) |
| *rnt-1* | The Runx transcription factor crucial to regulate the balance between seam cell proliferation and differentiation, promoting the proliferative fate in posterior seam daughters. | (Kagoshima et al., 2007; Nimmo, Antebi, & Woollard, 2005; Xia, Zhang, Huang, Sun, & Zhang, 2007) |
| *cki-1* | A cyclin-dependent kinase inhibitor only expressed in the Y cell and believed to be required for Y to PDA transformation initiation. | (Richard et al., 2011) |
| *ceh-16* | Seam cell homeostatic control between differentiation and proliferation. *ceh-16* loss of function mutation will drive seam cells into differentiation. | (Huang, Tian, Xu, & Zhang, 2009) |
| *apr-1* | A member of the Wnt signaling pathway, suppressor of *ceh-16(lf)* mutations*.* | (Huang et al., 2009) |
| *mex-3* | Involved in germline fate maintenance. Mutation causes ectopic transdifferentiation of germ cells. | (Ciosk, DePalma, & Priess, 2006) |
| *gld-1* | Involved in germline fate maintenance. Mutation causes ectopic transdifferentiation of germ cells. | (Ciosk et al., 2006) |
| *mes-4* | Regulation of active chromatin states and the exclusion of the MES-2/MES-3/MES-6 chromatin repression complex from the autosomes. | (Fong, Bender, Wang, & Strome, 2002) |
| *dpy-30* | A nuclear protein essential early in embryogenesis for dosage compensation, believed to be involved in epigenetic regulation of transcription. | (Hsu, Chuang, & Meyer, 1995) |
| *egl-38* | Mutations cause additional transdifferentiation of a second rectal cell into a PDA neuron. | (Chamberlin et al., 1997; Jarriault et al., 2008) |
| *mab-9* | Mutations cause additional transdifferentiation of a second rectal cell into a PDA neuron. | (Chisholm & Hodgkin, 1989; Jarriault et al., 2008) |
| *bet-1* | Methylated histone binder, involved in cell fate maintenance. | (Shibata, Takeshita, Sasakawa, & Sawa, 2010) |
| *mys-1* | Member of the MYST family of histone acetyltransferases (MYST HATs) which regulates BET-1 and is believed to maintain cell fate. | (Shibata, Sawa, & Nishiwaki, 2014; Shibata et al., 2010) |
| *mys-2* | Member of the MYST family of histone acetyltransferases (MYST HATs) which regulates BET-1 and is believed to maintain cell fate. | (Shibata et al., 2014; Shibata et al., 2010) |
| *utx-1* | Downstream factor of *glp-1/*Notch signalling in the germline (personal communications B. Tursun and Ciosk), H3K27 demethylase. | Pers. comm. B. Tursun/R. Ciosk |
| *set-2* | A H3K4 methyltransferase required during the Y to PDA transdifferentiation. | (Zuryn et al., 2014) |
| *wdr-5.1* | A H3K4 methyltransferase required during the Y to PDA transdifferentiation. | (Zuryn et al., 2014) |
| *lin-53* | Involved in germline fate maintenance. Mutation renders germ cells plastic towards an induced neuronal differentiation. | (Tursun, Patel, Kratsios, & Hobert, 2011) |
| *sir-2.1* | A sirtuin that deacetylase telomeric histones and protect those homologues sequences from recombination events. | (Wirth et al., 2009) |

**References for Table S1**

Baugh, L. R., Wen, J. C., Hill, A. A., Slonim, D. K., Brown, E. L., & Hunter, C. P. (2005). Synthetic lethal analysis of Caenorhabditis elegans posterior embryonic patterning genes identifies conserved genetic interactions. *Genome Biol, 6*(5), R45. doi:10.1186/gb-2005-6-5-r45

Berry, L. W., Westlund, B., & Schedl, T. (1997). Germ-line tumor formation caused by activation of glp-1, a Caenorhabditis elegans member of the Notch family of receptors. *Development, 124*(4), 925-936. Retrieved from http://www.ncbi.nlm.nih.gov/pubmed/9043073

http://dev.biologists.org/content/develop/124/4/925.full.pdf

Chamberlin, H. M., Palmer, R. E., Newman, A. P., Sternberg, P. W., Baillie, D. L., & Thomas, J. H. (1997). The PAX gene egl-38 mediates developmental patterning in Caenorhabditis elegans. *Development, 124*(20), 3919-3928. Retrieved from http://www.ncbi.nlm.nih.gov/pubmed/9374390

http://dev.biologists.org/content/develop/124/20/3919.full.pdf

Chisholm, A. D., & Hodgkin, J. (1989). The mab-9 gene controls the fate of B, the major male-specific blast cell in the tail region of Caenorhabditis elegans. *Genes Dev, 3*(9), 1413-1423. Retrieved from http://www.ncbi.nlm.nih.gov/pubmed/2606353

http://genesdev.cshlp.org/content/3/9/1413.full.pdf

Ciosk, R., DePalma, M., & Priess, J. R. (2006). Translational regulators maintain totipotency in the Caenorhabditis elegans germline. *Science, 311*(5762), 851-853. doi:10.1126/science.1122491

Djabrayan, N. J., Dudley, N. R., Sommermann, E. M., & Rothman, J. H. (2012). Essential role for Notch signaling in restricting developmental plasticity. *Genes & development, 26*, 2386-2391. doi:10.1101/gad.199588.112

Fong, Y., Bender, L., Wang, W., & Strome, S. (2002). Regulation of the different chromatin states of autosomes and X chromosomes in the germ line of C. elegans. *Science, 296*(5576), 2235-2238. doi:10.1126/science.1070790

Fukushige, T., Brodigan, T. M., Schriefer, L. A., Waterston, R. H., & Krause, M. (2006). Defining the transcriptional redundancy of early bodywall muscle development in C. elegans: evidence for a unified theory of animal muscle development. *Genes Dev, 20*(24), 3395-3406. doi:10.1101/gad.1481706

Greenwald, I. (2005). LIN-12/Notch signaling in C. elegans. *WormBook*, 1-16. doi:10.1895/wormbook.1.10.1

Greenwald, I. S., Sternberg, P. W., & Horvitz, H. R. (1983). The lin-12 locus specifies cell fates in Caenorhabditis elegans. *Cell, 34*(2), 435-444. Retrieved from http://www.ncbi.nlm.nih.gov/pubmed/6616618

Hsu, D. R., Chuang, P. T., & Meyer, B. J. (1995). DPY-30, a nuclear protein essential early in embryogenesis for Caenorhabditis elegans dosage compensation. *Development, 121*(10), 3323-3334. Retrieved from http://www.ncbi.nlm.nih.gov/pubmed/7588066

http://dev.biologists.org/content/develop/121/10/3323.full.pdf

Huang, X., Tian, E., Xu, Y., & Zhang, H. (2009). The C. elegans engrailed homolog ceh-16 regulates the self-renewal expansion division of stem cell-like seam cells. *Dev Biol, 333*(2), 337-347. doi:10.1016/j.ydbio.2009.07.005

Jarriault, S., Schwab, Y., & Greenwald, I. (2008). A Caenorhabditis elegans model for epithelial-neuronal transdifferentiation. *Proceedings of the National Academy of Sciences of the United States of America, 105*, 3790-3795. Retrieved from http://www.ncbi.nlm.nih.gov/pmc/articles/PMC2268801/pdf/zpq3790.pdf

Kagias, K., Ahier, A., Fischer, N., & Jarriault, S. (2012). Members of the NODE (Nanog and Oct4-associated deacetylase) complex and SOX-2 promote the initiation of a natural cellular reprogramming event in vivo. *Proceedings of the National Academy of Sciences of the United States of America, 109*, 6596-6601. doi:10.1073/pnas.1117031109

Kagoshima, H., Nimmo, R., Saad, N., Tanaka, J., Miwa, Y., Mitani, S., . . . Woollard, A. (2007). The C. elegans CBFbeta homologue BRO-1 interacts with the Runx factor, RNT-1, to promote stem cell proliferation and self-renewal. *Development, 134*(21), 3905-3915. doi:10.1242/dev.008276

Germline proliferation and its control, WormBook.

Lambie, E. J., & Kimble, J. (1991). Two homologous regulatory genes, lin-12 and glp-1, have overlapping functions. *Development, 112*(1), 231-240. Retrieved from http://www.ncbi.nlm.nih.gov/pubmed/1769331

http://dev.biologists.org/content/develop/112/1/231.full.pdf

Nimmo, R., Antebi, A., & Woollard, A. (2005). mab-2 encodes RNT-1, a C. elegans Runx homologue essential for controlling cell proliferation in a stem cell-like developmental lineage. *Development, 132*(22), 5043-5054. doi:10.1242/dev.02102

Priess, J. R. (2005). Notch signaling in the C. elegans embryo. *WormBook*, 1-16. doi:10.1895/wormbook.1.4.1

Richard, J. P., Zuryn, S., Fischer, N., Pavet, V., Vaucamps, N., & Jarriault, S. (2011). Direct in vivo cellular reprogramming involves transition through discrete, non-pluripotent steps. *Development, 138*, 1483-1492. doi:10.1242/dev.063115

Shibata, Y., Sawa, H., & Nishiwaki, K. (2014). HTZ-1/H2A.z and MYS-1/MYST HAT act redundantly to maintain cell fates in somatic gonadal cells through repression of ceh-22 in C. elegans. *Development, 141*(1), 209-218. doi:10.1242/dev.090746

Shibata, Y., Takeshita, H., Sasakawa, N., & Sawa, H. (2010). Double bromodomain protein BET-1 and MYST HATs establish and maintain stable cell fates in C. elegans. *Development, 137*(7), 1045-1053. doi:10.1242/dev.042812

Tursun, B., Patel, T., Kratsios, P., & Hobert, O. (2011). Direct conversion of C. elegans germ cells into specific neuron types. *Science, 331*, 304-308. doi:10.1126/science.1199082

Unhavaithaya, Y., Shin, T. H., Miliaras, N., Lee, J., Oyama, T., & Mello, C. C. (2002). MEP-1 and a homolog of the NURD complex component Mi-2 act together to maintain germline-soma distinctions in C. elegans. *Cell, 111*(7), 991-1002. Retrieved from http://www.ncbi.nlm.nih.gov/pubmed/12507426

http://ac.els-cdn.com/S0092867402012023/1-s2.0-S0092867402012023-main.pdf?_tid=7fed0336-56f3-11e5-ae8c-00000aab0f02&acdnat=1441804132_6f013250669311e52e3af7694fe00d89

Wirth, M., Paap, F., Fischle, W., Wenzel, D., Agafonov, D. E., Samatov, T. R., . . . Jedrusik-Bode, M. (2009). HIS-24 linker histone and SIR-2.1 deacetylase induce H3K27me3 in the Caenorhabditis elegans germ line. *Mol Cell Biol, 29*(13), 3700-3709. doi:10.1128/MCB.00018-09

Xia, D., Zhang, Y., Huang, X., Sun, Y., & Zhang, H. (2007). The C. elegans CBFbeta homolog, BRO-1, regulates the proliferation, differentiation and specification of the stem cell-like seam cell lineages. *Dev Biol, 309*(2), 259-272. doi:10.1016/j.ydbio.2007.07.020

Zuryn, S., Ahier, A., Portoso, M., White, E. R., Morin, M. C., Margueron, R., & Jarriault, S. (2014). Transdifferentiation. Sequential histone-modifying activities determine the robustness of transdifferentiation. *Science, 345*(6198), 826-829. doi:10.1126/science.1255885
