# Supplementary material for: Polycomb and Notch signaling regulate cell proliferation potential during Caenorhabditis elegans life cycle
Source: Life Sci Alliance. 2018 Dec 26;2(1):e201800170. doi: 10.26508/lsa.201800170 (PMC6306570; doi:10.26508/lsa.201800170)
Supplement: Supplementary file 2 [file LSA-2018-00170_TableS2.docx]

**Strains list**

| Strain | International code | Genotype |
| --- | --- | --- |
| PMW3 | JJ1271 or GW36 | *glo-1(zu391) X.* |
| PMW4 | GW76 | *gwIs4[baf-1::GFP-lacI;myo-3::RFP] X.* |
| PMW48 | GW429 | *gwIs59[pha-4::mCherry; unc-119(+); 256xLacO;4xLexA] gwIs39[baf-1::GFP-LacI::let-858 3'UTR; vit-5::GFP] III. unc-119(?) III* |
| PMW200 |  | *rrrSi261[myo-3p::cey-4::SL2::gfp:h2b; unc-119] I. ubsSi13[hsp-16.2p::hlh-1::SL2::mCherry] II. unc-119(ed3) III.* |
| PMW217 |  | *rrrSi261 I. ubsSi14[hsp-16.2p::end-1::SL2::mCherry] II. unc-119(ed3) III.* |
| PMW218 |  | *rrrSi261 I. gwSi3[hsp16.3p::mCherry] II. unc-119(ed3) III.* |
| PMW262 |  | *rrrSi261 I. ubsSi13 II. unc-119(ed3) met-2(n4256) set-25(n5021) III.* |
| PMW283 |  | *rrrSi261 I. gwSi3 II. unc-119(ed3) met-2(n4256) set-25(n5021) III.* |
| PMW284 |  | *rrrSi261 I. mes-2(bn11) unc-4(e120) / mnC1 II.* |
| PMW285 |  | *rrrSi261 I. ubsSi13 mes-2(bn11) unc-4(e120) / mnC1 II. unc-119(ed3) III.* |
| PMW292 |  | *ubsSi13 II. unc-119(ed3) III. gwIs4 X.* |
| PMW325 | GW828 | *cec-4(ok3124) IV.* |
| PMW329 |  | *rrrSi261 I. ubsSi13 II. cec-4(ok3124) IV.* |
| PMW331 |  | *rrrSi261 I. gwSi3 II. cec-4(ok3124) IV.* |
| PMW392 |  | *rrrSi261 I. ubsSi14 mes-2(bn11) unc-4(e120) / mnC1 II. unc-119(ed3) III.* |
| PMW426 |  | *rrrSi261 I. mes-2(bn11) unc-4(e120) / mnC1 nIs190 II.* |
| PMW427 |  | *rrrSi261 I. ubsSi13 mes-2(bn11) unc-4(e120) / mnC1 nIs190 II. unc-119(ed3) III.* |
| PMW428 |  | *rrrSi261 I. ubsSi14 mes-2(bn11) unc-4(e120) / mnC1 nIs190 II. unc-119(ed3) III.* |
| PMW476 |  | *rrrSi261 I. ubsSi13 II. lin-12(n950) III.* |
| PMW504 |  | *rrrSi261 I. ubsSi13 mes-2(bn11) unc-4(e120) / mnC1 II. unc-119(ed3)(?) III. glo-1(zu391) X.* |
| PMW517 |  | *rrrSi261 I. mes-2(bn11) unc-4(e120) / mnC1 II. glo-1(zu391) X.* |
| PMW527 |  | *rrrSi261 I. gwSi3 II. lin-12(n950) III.* |
| PMW528 |  | *rrrSi261 I. ubsSi13 mes-2(bn11) unc-4(e120) / mnC1 II. unc-119(ed3) III.* *ubs5[lin-12(n137)] III.* |
| PMW537 |  | *rrrSi261 I. ubsSi13 mes-2(bn11) unc-4(e120) / mnC1 II. unc-119(ed3)(?) III. gwIs59 gwIs39 III.* |
| PMW538 |  | *rrrSi261 I. mes-2(bn11) unc-4(e120) / mnC1 II. gwIs59 gwIs39 III.* |
| PMW549 | PD4666 | *ayIs6 [hlh-8::GFP fusion + dpy-20(+)] X.* |
| PMW550 | JR667 | *unc-119(e2498::Tc1) III. wIs51 [SCMp::GFP + unc-119(+)].* |
| PMW558 |  | *mes-2(bn11) unc-4(e120) / mnC1 II. ayIs6 X.* |
| PMW559 |  | *ubsSi13 mes-2(bn11) unc-4(e120) / mnC1 II. unc-119(ed3)(?) III. ayIs6 X* |
| PMW560 |  | *mes-2(bn11) unc-4(e120) / mnC1 II. wIs51.* |
| PMW561 |  | *ubsSi13 mes-2(bn11) unc-4(e120) / mnC1 II. unc-119(ed3)(?) III. wIs51.* |
| PMW580 |  | *unc-119(ed3) III. icbSi1[arf-3:pes-10::mCherry::H2B::unc-54 3'UTR+cb-unc-119].* |
| PMW592 |  | *rrrSi261 I. mes-2(bn11) unc-4(e120) / mnC1 II. unc-119(ed3)(?) III. icbSi1.* |
| PMW600 |  | *rrrSi261 I. ubsSi13 mes-2(bn11) unc-4(e120) / mnC1 II. unc-119(ed3)(?) III. icbSi1.* |
| PMW614 | VT825 | *dpy-20(e1282) IV. mals113[cki-1::GFP + dpy-20(+)]* |
| PMW638 |  | *mes-2(bn11) unc-4(e120) / mnC1 II. dpy-20(e1282)(?) IV. mals113.* |
| PMW639 |  | *ubsSi13 mes-2(bn11) unc-4(e120) / mnC1 II. unc-119(ed3) III. dpy-20(e1282)(?) IV. mals113.* |
| PMW656 | OH10689 | *otIs355 [rab-3p(prom1)::2xNLS::TagRFP] IV.* |
| PMW666 |  | *rrrSi261 I. ubsSi14 mes-2(bn11) unc-4(e120) / mnC1 II. icbSi1.* |
| PMW673 |  | *rrrSi261 I. mes-2(bn11) unc-4(e120) / mnC1 II. otIs355 IV.* |
| PMW675 |  | *rrrSi261 I. ubsSi13 mes-2(bn11) unc-4(e120) / mnC1 II. unc-119(ed3)(?) III. otIs355 IV.* |
| PMW685 | JK2868 | *qls56 [lag-2p::GFP + unc-119(+)] V.* |
| PMW689 |  | *rrrSi261 I. mes-2(bn11) unc-4(e120) / mnC1 II. unc-119(ed3)(?) III. icbSi1. qls56 V.* |
| PMW694 |  | *rrrSi261 I. ubsSi13 mes-2(bn11) unc-4(e120) / mnC1 II. unc-119(ed3)(?) III. icbSi1. qls56 V.* |
| PMW711 |  | *rrrSi261 I. ubsSi13 II. unc-119(ed3)(?) III. qls56 V.* |
| PMW720 | *VT765* | *unc-36(e251) III; maIs103 [rnr::GFP + unc-36(+)].* |
| PMW724 |  | *rrrSi261 I. ubsSi14 mes-2(bn11) unc-4(e120) / mnC1 II. unc-119(ed3)(?) III. gwIs59 gwIs39 III.* |
| PMW728 |  | *ubsSi13 mes-2(bn11) unc-4(e120) / mnC1 II. unc-119(ed3)(?) III. maIs103.* |
| N2 |  | Wild-type Bristol strain |
