# Supplementary material for: Polycomb and Notch signaling regulate cell proliferation potential during Caenorhabditis elegans life cycle
Source: Life Sci Alliance. 2018 Dec 26;2(1):e201800170. doi: 10.26508/lsa.201800170 (PMC6306570; doi:10.26508/lsa.201800170)
Supplement: Supplementary file 3 [file LSA-2018-00170_Supplementary_Materials_and_Methods.doc]

Materials and Methods

**Synchronization of *mes-2* animals**

*mes-2* animals are sterile in the second generation. Synchronized F2 animals were prepared by manually picking F1 homozygotes (phenotypically Unc) from balanced parents (wild-type). F2 homozygotes were obtained as for the wild-type animals. For TF induction, synchronized worms were washed off the plates with M9, transferred to an Eppendorf tube, spun at 1000 g for 1 minute and washed once before spinning them again. The supernatant was then aspirated to concentrate the worms in a small volume (~20-50µL).

**Oxidative stress**

The oxidative stress was performed as in(Kumsta et al. 2011). Synchronized L1 worms were placed on NG2 plates with bacteria. After three hours they were collected by centrifugation in M9 medium. One hundred microliters of worms were added to 2ml of M9 with 1 mM, 6 mM, 10 mM H_2_O_2_. The tubes were left for 30 minutes at room temperature in a rotating roller drum. The worms were collected by centrifugation and washed with M9 medium. The supernatant was aspirated, the animals were heat-shocked for 30 minutes in a 33°C water bath and transferred to a fresh plate seeded with OP50 and incubated at 22.5°C.

**Osmotic stress**

NG2 plates were made with a high concentration of NaCl (1M). They were seeded with OP50 one day before to minimize the variation in salt concentration due to evaporation (as in (Wheeler and Thomas 2006)). Synchronized worms were placed on plates with NaCl 1M for one hour. The worms were collected from the plate with M9. After centrifugation and aspiration of the supernatant the heat shock was performed in 33°C water bath for 30 minutes. Larvae were transferred on a fresh plate with OP50 with the standard concentration of 50 mM of NaCl.

Kumsta C, Thamsen M, Jakob U. 2011. Effects of oxidative stress on behavior, physiology, and the redox thiol proteome of Caenorhabditis elegans. *Antioxid Redox Signal* **14**: 1023-1037.

Wheeler JM, Thomas JH. 2006. Identification of a novel gene family involved in osmotic stress response in Caenorhabditis elegans. *Genetics* **174**: 1327-1336.
